# Molecular identification, characterization and antibacterial activity of fungal-mediated silver nanoparticles against *Bacillus subtilis* sh3 and *Klebsiella pneumoniae* sh4

**DOI:** 10.1038/s41598-026-42107-9

**Published:** 2026-03-29

**Authors:** Mahmoud AbdEl-Mongy Ismail, Shimaa Rafat, Hanafy A Hamza, A. B. Abeer Mohammed

**Affiliations:** Department of Microbial Biotechnology, Faculty of Biotechnology - University of Sadat City, Sadat City, Egypt

**Keywords:** Fusarium oxysporum, Silver nanoparticles, Biosynthesis, Multidrug resistance, Antibacterial, Cytotoxicity, Biochemistry, Biological techniques, Biotechnology, Drug discovery, Microbiology

## Abstract

The increasing prevalence of multidrug-resistant bacteria has created a determined need for new antimicrobial approaches. In the present study, *Fusarium oxysporum* SH1 was utilized as a biological source for the green synthesis of silver nanoparticles through its extracellular metabolites. The produced nanoparticles were confirmed to form within the nanoscale range and exhibited stable physicochemical characteristics. They showed clear antibacterial activity against two multidrug-resistant clinical isolates, *Bacillus subtilis* sh3 and *Klebsiella pneumoniae* sh4, with low minimum inhibitory concentrations. In addition, combining the nanoparticles with commonly used antibiotics enhanced the inhibition zones compared with the use of either agent alone, indicating a measurable improvement in antibacterial performance. The biosynthesized nanoparticles also demonstrated selective cytotoxic effects when tested on human cell lines. MCF7 breast cancer cells exhibited a markedly lower IC_50_ value than normal HFB4 melanocytes, and treatment was associated with distinct morphological alterations indicative of compromised cell structure. These findings indicate a stronger growth-inhibitory effect on malignant cells under the tested conditions. Generally, this study presents a straightforward and eco-friendly method for producing biologically active silver nanoparticles using *F. oxysporum* SH1. The nanoparticles displayed dual biological functions as antibacterial activity against resistant pathogens and selective cytotoxicity toward cancer cells. These experimentally supported observations suggest that fungal-mediated silver nanoparticles represent a promising candidate for further investigation in antimicrobial and anticancer applications.

**Supplementary Information:**

The online version contains supplementary material available at 10.1038/s41598-026-42107-9.

## Introduction

One of the most important issues facing the medical field today is the developing bacterial resistance to antimicrobial medications, which poses a major risk to human health. Over time, the antibiotics that are mostly used to treat bacterial diseases either develop flaws or cause strains of bacteria to become resistant to them^1^. Therefore, it becomes imperative to look for options that aid in overcoming such obstacles. According to^2^. Numerous nanomaterials that employ different metal ions, including copper, magnesium, gold, and silver, can create these structures. One of the most important issues facing the medical field today is the developing bacterial resistance to antimicrobial medications, which poses a major risk to human health. Over time, nanoparticles have been successfully employed in a number of medical and pharmaceutical uses. The production of silver nanoparticles (AgNPs) by microbiological synthesis is more rapid, lower cost, and more ecologically friendly than chemical synthesis. Approaches have attracted increased attention in recent years^3^. In general, The scientific community is particularly interested in the development of nanoparticles (NPs) because of their special qualities and technological uses, which help a number of sectors, including the pharmaceutical and cosmetics industries., industry, energy, and agriculture^4^.Scientists are particularly interested in metal nanoparticles (NPs) owing to their physicochemical properties, which include large surface area, precise pore size, and strong reactivity, which allow them to cross The distinction between bulk and atomic structures^5^.Silver is one of the noble metals that is used extensively in medicine since Its antimicrobial and wound-healing qualities have been demonstrated. Research indicates that the colloidal form of silver nanoparticles (AgNPs) has good conductivity, chemical stability, and antibacterial and catalytic properties^6^. Numerous techniques, including physical, chemical, biological, and further electrochemical, photochemical, radiolytic, and sonolytic processes approaches, can be used to manufacture AgNPs^7^. Biological synthesis has emerged as an effective approach for producing silver nanoparticles, as naturally derived capping agents enhance nanoparticle stability and prolong their shelf life. Green biosynthesis also offers practical advantages such as low-cost downstream processing and efficient purification. Microorganisms including fungi and bacteria as well as various plant extracts, represent the primary biological sources used for AgNPs production^8^. There have been reports of additional sources for AgNP production that use biological materials such egg white^9^, milk^10^, honey, and coconut water. The synthesis, characterization, and biological characteristics of silver nanoparticles (AgNPs) were described by Otunola and Afolayan using silver nitrate solution and extracts from garlic, ginger, and cayenne pepper (1:1: 1; w/w/w). According to the World Health Organization Global Antimicrobial Resistance and Use Surveillance System (GLASS) report (WHO, 2022), antimicrobial resistance is increasing worldwide, threatening the efficacy of several commonly used antibiotics^11^. When bacteria that are resistant to more than two antimicrobial classes show in vitro resistance to one or more medications, they are responsible for about 70% microbiological illnesses. Rapid bacterial genetic alterations, decreased membrane penetration, drug inactivation or enzymatic degradation, and target protein modification in pathogens that cause infectious illnesses could all be responsible for this^12^. Although the green synthesis of silver nanoparticles using *Fusarium oxysporum* has been previously described, the present study introduces several distinct aspects. The SH1 strain used here has not been reported before for nanoparticle production, and it generated particles with smaller size and higher stability than earlier formulations. In addition, the work combines antibacterial testing against recently isolated multidrug-resistant strains with a parallel evaluation of selective cytotoxicity toward cancer cells, providing a broader biological assessment than what is commonly available in literature.

The current study used the cell free exometabolites of *Fusarium oxysporum* as a biocatalyst to create tiny silver nanoparticles in an environmentally acceptable manner. SNPs’ antioxidant and anticancer properties, as well as their biological mechanism of action against bacteria, were examined. The capacity of a broad variety of filamentous fungus and yeasts, including *Phoma sp* to produce silver nanoparticles has also been documented^13^. *Aspergillus sp. Fusarium oxysporum*^14^. Additionally, Madbouly et al. employed Chaetomium globosum to produce AgNPs that show antifungal activity in vivo against *Fusarium oxysporum*, which causes tomato wilt. Every fungus species has a unique capacity to provide NPs a range of physicochemical and biological characteristics, which may be connected to their capacity to generate a large number of important metabolites.

## Materials and methods

This inquiry was carried out in microbial biotechnology department, Genetic Engineering and Biotechnology Research Institute, university of Sadat city, Sadat city, Egypt.

### Chemicals

Silver nitrate, five antibiotic discs (Oxoid, UK) were chosen to assess the sample’s antimicrobial susceptibility: Streptomycin (S, 10 µg), Ciprofloxacin (CIP, 5 µg), Erythromycin (E, 15 µg), ceftriaxone (CTR, 30 µg), and aztreonam (AT, 30 µg). in Egypt and sterile distilled water was used throughout the experiments.

### Microbial media

When a straightforward procedure was employed, Czapek’s-Dox agar (CDA) medium, Sabouraud Dextrose Agar, and Nutrient Agar were used. These techniques are still frequently employed in bacteriological investigations of material selection and are also advised by standard methods. Nutrient broth is a non-selective approach for the predictable culture of microorganisms that was originally intended for use in the Standard Technique^15^.

### Fungal strain isolation and silver nanoparticle biosynthesis

Using Czapek’s-Dox agar (CDA) medium, the fungal isolates utilized in this investigation were isolated from a variety of spoiled tomato samples that were gathered from the Suez Governorate in Egypt. For additional research, the fungal isolates produced were collected, purified by re-inoculation onto CDA medium, and then stored onto the Sabouraud Dextrose Agar medium, which contained (g/l) 10 g pepton, 20 g glucose, and 20 g Agar^16^. This fungal isolate’s potential for SNP biosynthesis was investigated using a modified medium that contained (g/l): 0.5 MgSO_4_.7H_2_O, 0.01 was included as part of the modified Czapek-based medium because magnesium ions support fungal metabolic functions and help maintain the enzymatic activity involved in extracellular nanoparticle formation. After incubation at 28°C for 5 days and under sterile conditions, the mycelium was harvested by filtration from a medium and washed thoroughly using sterile distilled water. Following five days of sterile incubation at 28°C, the mycelium was extracted by filtering it out of a medium and completely cleaned with sterile distilled water. After thoroughly mixing the 5.0 g of fungal mycelium with 50 ml of deionized water, it was incubated under the previously mentioned conditions. After filtering, the mycelium was centrifuged. Then, 0.017 mg of AgNO_3_ was added to the free-cell filtrate. Following five days of dark incubation at 28°C and 100 rpm, the biosynthetic SNPs were collected by centrifugation at 8,000 rpm for ten minutes. Several centrifugation cycles were carried out for 20 minutes at 8,000 rpm in order to retrieve the green generated SNPs. The production yield of AgNPs was estimated based on the dry weight of the recovered nanoparticles. Following the protocol of^17^, the synthesized AgNPs were collected by centrifugation at 10,000 rpm, washed thoroughly with deionized water, and dried to obtain a constant weight. The yield was found to be approximately 13.5 mg/100 mL, indicating a high bio-reduction efficiency of the fungal filtrate.” Parallel to this, the identical conditions were used for the blank (fungal supernatant without AgNO_3_) and negative control (silver nitrate alone)^18^. The formation of silver nanoparticles was monitored visually by the change from light yellow to pale purple, indicating the reduction of silver ions, and was further confirmed using UV-Vis spectroscopy^19^.

### Molecular identification of the most potent fungal isolate synthesizing SNPs

Following morphological, cultural, and microscopical examination, the fungal isolate was identified using the usual keys^20,21^. The prospective SNPs producer was molecularly identified using the whole internal transcribed sequence (ITS) of the rDNA region. The genomic DNA was extracted and amplified using ITS1 (5’-TCC GTA GGT GAA CCT GCG G-3’) and ITS4 (5’-TCC TCC GCT TAT TGA TAT GC-3’) primers. 2 µl extracted genomic DNA, 1 µl of each primer,4.5 µl PCR grade water, and 12.5 µl (2× PCR master mixture (AlphaDNA Co, Canada) makes up the (25 µl) PCR mixture^22^. A T3 Thermal Cycler (Biometra) was used to conduct the PCR. with 5 min of hot beginning at 95 °C, 35 cycles of 95 °C for 30 s of annealing at 56 °C, 50 s of extension at 72 °C, and 10 min of final extension at 72 °C. The same primer sets were used to sequence the PCR amplicons after they had been examined on a 1% agarose gel. The BLAST tool was used to match the acquired sequence with the ITS sequences in the database of GenBank in order to determine the fungal isolate’s phylogenetic position. The DNA was imported into ClustalW for multiple sequence alignment.

### The biosynthetic silver nanoparticles’ characteristics

#### Analysis of UV–Vis spectroscopy

The formation of silver nanoparticles was examined by UV-vis spectroscopy. Sample preparation involved diluting the nanoparticle suspension with distilled water at a ratio of 1:10. The absorption spectrum was recorded using a UV-visible spectrophotometer (GBC model 932 AA) equipped with a 1 cm path length quartz cuvette. Measurements were performed at room temperature (25 °C), and the wavelength scan was carried out in the range of 200–800 nm with a scanning speed of 200 nm/min and a resolution of 1 nm. Distilled water was used as a blank reference to baseline the instrument before each measurement.

#### Fourier-Transform Infrared (FT-IR)

The samples were freeze-dried to obtain a dry powder. For sample preparation, approximately 2.0 mg of the dried nanoparticles were mixed with 300.0 mg of potassium bromide (KBr) in an agate mortar to ensure homogeneous distribution. The mixture was then compressed into a transparent pellet using a hydraulic press at a pressure of 10 tons for 2 min. FT-IR spectra were recorded using a JASCO FTIR-6200 spectrophotometer (JASCO Corporation, Japan). The instrument was equipped with a high-intensity ceramic light source and a DLATGS detector. The obtained infrared spectra were scanned using JASCO instrument software and the results are tabulated against the wave number for each sample. Spectra were collected in the wavenumber range of 400–4000 cm^− 1^ with a spectral resolution of 0.25 cm^− 1^^23,24^.

#### Silver nanoparticle TEM analysis

The morphology, size, and internal structure of the biosynthesized silver nanoparticles were examined using transmission electron microscopy following the method described by^25^, For sample preparation, samples suspension was first diluted with distilled water (1:20 v/v) to achieve an appropriate concentration for imaging. A single drop (approximately 10 µL) of the diluted nanoparticle dispersion was placed onto a carbon-coated copper grid (300 mesh) and allowed to air-dry at room temperature for 24 h to ensure complete solvent evaporation.

TEM imaging was performed using a JEOL transmission electron microscope (JEOL Ltd., Tokyo, Japan) operated at an accelerating voltage of 200 kV.

#### Assessment of scanning electron microscopy (SEM)

The surface morphology and particle distribution of the biosynthesized silver nanoparticles (AgNPs-Fs) were investigated using scanning electron microscopy according to the method described by^26^. For sample preparation, a small amount of the dried AgNP powder was dispersed in distilled water by ultrasonication for 5 min to obtain a homogeneous suspension. A drop of approximately 50 µL of the nanoparticle suspension was carefully placed onto aluminum specimen stubs using a micropipette. The sample was secured with double-sided carbon adhesive tape and allowed to dry completely at room temperature overnight. SEM imaging was carried out using a JEOL JSM-5500LV scanning electron microscope (JEOL Ltd., Tokyo, Japan) operated at an accelerating voltage of 20 kV. The size distribution and morphological characteristics of the nanoparticles were analyzed from the obtained micrographs.

#### Analysis using X-ray diffraction (XRD)

The crystalline structure and phase composition of the biosynthesized silver nanoparticles were investigated using X-ray diffraction analysis following the procedure described by^27^. The lyophilized AgNPs-Fs powder was homogenized using a ball mill, then pressed onto the sample holder with a glass slide to create a uniform surface. XRD measurements were performed using an (X Pert PRO) diffractometer (PANalytical, The Netherlands) equipped with a Cu-Kα radiation source (λ = 1.54184 Å) operated at 40 kV and 30 mA. Diffraction patterns were recorded at room temperature over a 2θ range of 5–60° with a step size of 0.02°. The crystallite size was calculated using the Debye-Scherrer equation, which reads D = Kλ/βcosθ.

where λ is the X-ray wavelength (1.5418 Å), β is the full width at half maximum (FWHM) of the diffraction peak in radians, θ is the Bragg angle, D is the average crystallite size (nm), and K is the shape factor (0.9).

#### Dynamic Light Scattering (DLS) Measurement

Dynamic Light Scattering (DLS) analysis was employed to assess the size distribution and average hydrodynamic diameter of the biosynthesized silver nanoparticles (AgNPs-Fs). To prevent multiple scattering effects, the nanoparticle solution was suitably diluted with deionized water before testing. A Malvern Zetasizer Nano ZS device with a 633 nm laser was used to do the measurements at 25 °C^28^. To guarantee the accuracy and consistency of the findings, each sample was examined three times. The intensity-weighted average particle size was obtained by automatically processing the data using instrument software.

#### EDAX, or energy-dispersive X-ray analysis

Using Energy-Dispersive X-ray Analysis (EDAX), the biosynthesis’s elemental composition silver nanoparticles (AgNPs-Fs) were identified. Using double-sided carbon adhesive tape, the samples were then sputter-coated with a thin gold layer using an SPI-Module sputter coater to ensure electrical conductivity. EDX analysis was performed using an Oxford 6587 INCA X-sight energy-dispersive X-ray microanalyzer attached to a JEOL JSM-5500LV scanning electron microscope, operated at an accelerating voltage of 20 kV. The instrument software automatically determined the relative weight and atomic percentages of the discovered elements based on the recording of spectra over a broad energy range.

### Bacterial cultures

#### Sample collection

Stool samples taken from the laboratory in Suez, Egypt, were used to isolate bacteria. The current study excluded patients who had received antibiotic treatment during the last three days. The samples were delivered under aseptic conditions to Sadat City University’s Microbiology Lab (Genetic Engineering Biotechnology Research Institute) in Egypt. The bacterial cultures that were created were cultivated until they reached the mid-log phase. Every chemical was of analytical quality and was acquired by Sigma-Aldrich. The bacterial samples were again put on LB agar medium, which had a pH of 7 and contained 1.5% agar, 0.5% yeast extract, 0.5% NaCl, and 1% peptone. Purified isolates were obtained in this way, and they were subsequently streaked and evaluated.

#### The molecular composition of the multidrug-resistant bacteria

Based on their 16 S-rRNA gene sequencing, a specific group of multidrug-resistant bacteria, namely sh3 and sh4, were found. Colony PCR was performed using the primers of 27 F (5′-AGA GTT TGA TCC TGG CTC AG-3′) and 1492R (5-CGG TTA CCT TGT TAC GAC TT -3′) to extract the bacterial genomic DNA and use it as a template for amplification^29,30^. The T3 Thermal Cycler (Biometra) was used to perform the PCR. The PCR products were purified and sequenced using the forward and reverse primers. The BLAST tool was then used to compare the resultant sequences with the NCBI database. 6 µl of extracted genomic DNA, 1 µl of each primer, 12.5 µl of Emerald Amp GT PCR master mix (2x premix), and 4.5 µl of PCR grade water make up the 25 µl PCR mixture. The PCR was started for 5 min at 94 °C, followed by 35 cycles of 94 °C for 30 s, annealing at 56 °C for 40 s, extension at 72 °C for 50 s, and finally a final extension at 72 °C for 10 min. In order to assess the PCR mixture, it was run on an agarose gel, stained with ethidium bromide, and examined under ultraviolet (UV) light. The QIA fast PCR purification kit was used to purify the amplicon once more. (Germany, the Netherlands, Qiagen).

#### Construction of phylogenetic trees

The Sanger Sequencing Technology was utilized to sequence the 16 S rRNA gene fragment bidirectionally with the ABI 3730XL DNA Analyzer from Applied Biosystems (Elim biopharmaceutical, USA). The Blast tool was applied to examine the DNA resemblances at http://blast.ncbi.nlm.nih.gov/Blast.cgi. Both molecular phylogeny and multiple sequence alignment were performed using Bio Edit software from Informer Technologies, Inc. GenBank received the produced sequence and assigned it the accession numbers PX210416.1 and PX210417.1. The aligned sequence from the BLAST analysis was used. The phylogenetic tree was constructed using the free online program ClustalW and the 16 S rRNA gene sequences in comparison to the other closely related gene sequences.

#### Determination of Minimum Inhibitory Concentration (MIC) of AgNPs-Fs

The antibacterial activity of the biosynthesized AgNPs-Fs was preliminarily evaluated using the agar disk-diffusion method. Although the CLSI-recommended broth microdilution method is the standard for MIC determination, disk diffusion was used to assess the inhibitory potential. The zone diameters were measured, and the MIC values were interpreted based on previous studies of similar silver nanoparticles^31,32^. To determine the minimum inhibitory concentration (MIC), sterile paper discs (6 mm) were loaded with 50 µL of AgNPs-Fs at serial concentrations (0.17, 0.085, 0.042, 0.021, 0.01, 0.005, 0.002, and 0.001 mg/mL).The initial concentration of AgNPs was 5 mg/ml and serial dilution 14 times occurred and each concentration tested by disk diffusion test. and placed on nutrient agar plates inoculated with standardized bacterial suspensions (1 × 10^5^ CFU/mL) of *Bacillus subtilis* sh3 and *Klebsiella pneumoniae* sh4. After incubation at 37 °C for 24 h, inhibition zone diameters were recorded, and the lowest concentration producing a visible inhibition zone was considered the effective MIC under disk-diffusion conditions. All assays were performed in triplicate, and the mean ± standard deviation (SD) was calculated.

#### Test for antibiotic susceptibility

The antibiotic resistance of the test bacterial strains was evaluated using the traditional disc diffusion method, which was described by^33^. Five commercial antibiotic discs (Oxoid, UK) were selected: streptomycin (S, 10 µg), ciprofloxacin (CIP, 5 µg), erythromycin (E, 15 µg), ceftriaxone (CTR, 30 µg), and aztreonam (AT, 30 µg). The diameters of the inhibition zones on the inoculation plates were measured in millimeters following a 24-hour incubation period at 37 °C. The standards set by the Clinical and Laboratory Standards Institute’s criteria^34^.

#### Antibacterial assessment

As recommended by^35^, Biosynthesized silver nanoparticles’ antibacterial and antifungal activity was examined using the agar well-diffusion method made by the fungus *Fusarium oxysporium* SH1 using a variety of Gram-positive *Bacillus subtilis* sh3 and Gram-negative bacteria *Klebsiella pneumoniae* sh4. Discs containing the chosen antibiotics were impregnated with biosynthesized silver nanoparticles (AgNPs) before being placed on inoculated agar plates in order to evaluate the potential *interaction* between the two substances. The incubation conditions for the plates were identical to those used for the conventional susceptibility test. The sizes of the inhibitory zones were measured and compared to those obtained with AgNPs or antibiotics alone^36^.

### In vitro cytotoxic and selective anti-proliferative

#### Cell lines and culture conditions

The National Cancer Institute (Cairo, Egypt) provided the normal human melanocyte cell line HFB4 and the human breast cancer cell line MCF7. Both cell types were cultured in Roswell Park Memorial Institute (RPMI) 1640 medium, which was boosted by 1% L-glutamine, 1% penicillin-streptomycin, and 2% fetal bovine serum (FBS). The cultures were housed in a humidified environment with 5% CO_2_ at 37 °C. To make sure the cells stayed in the exponential development phase, subculturing was done every two to three days.

#### Cytotoxicity Determination via MTT Assay

Used the MTT colorimetric assay according to^37^, examine the cytotoxic potential of silver nanoparticles made with Fusarium sp. (AgNPs-Fs) against the MCF7 and HFB4 cell lines. To put it briefly, cells were cultivated in 96-well plates at a density of 1 × 10^5^ cells/mL (100 µL/well), and they were incubated for 24 h to allow for adhesion. The cells were subjected to two-fold repeated dilutions of AgNPs-Fs produced in RPMI containing 2% FBS after being gently washed with PBS after monolayer formation. Three untreated control wells were used. 20 µL of MTT solution (5 mg/mL in PBS) was added to each well after 48 h of incubation, and the wells were then incubated for a further 4 h. After removing the supernatant, the generated formazan crystals were dissolved using 200 µL of dimethyl sulfoxide (DMSO). A microplate reader was used to detect absorbance at 560 nm. Using GraphPad Prism software, nonlinear regression analysis of dose-response curves was used to determine the IC_50_ values and calculate the percentage of cells that were viable. According to^38^, the selectivity index (SI) was calculated as SI=IC_50_ (MCF7)/IC_50_ (HFB4).

#### Morphological assessment

Under an inverted phase-contrast microscope, treated cells were viewed to visualize cytotoxic effects. Shrinkage, rounding, detachment from the substrate, and disruption of monolayer integrity were among the morphological changes observed, and these characteristics were contrasted with untreated controls to verify the presence of cell damage^39^.

### Statistical analysis

All experiments were performed in triplicate, and the results are expressed as mean ± standard error. The data were checked for normality and then analyzed using one-way ANOVA to compare the treatments. When significant differences were detected (*p* < 0.05), a post-hoc test was applied to separate the means. Different letters (a, b, c, d) above the bars indicate statistically significant difference.

## Results and discussion

### Screening and identification of used fungi

**Fig. 1:**
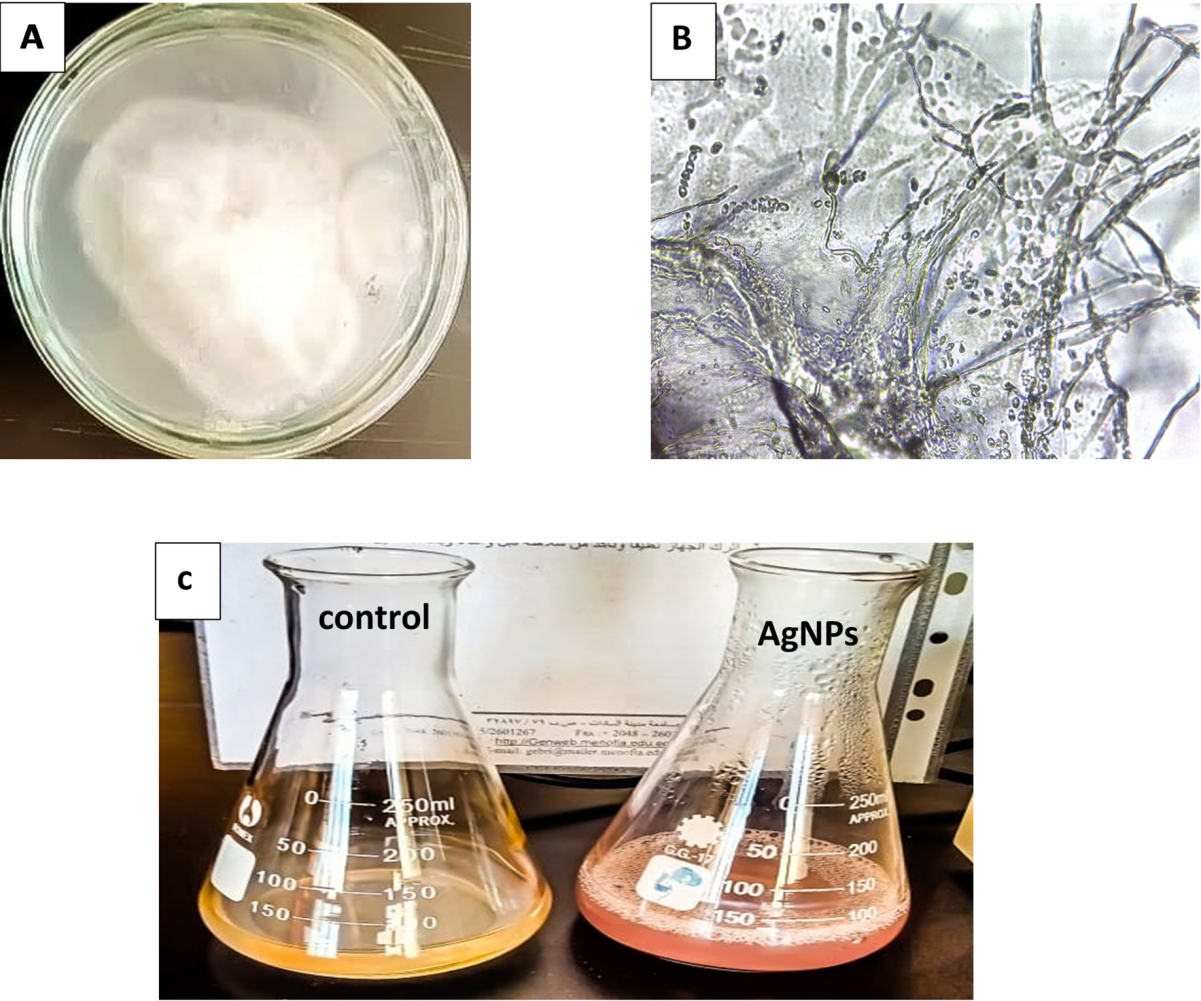
(**A**), Morphological of *Fusarium oxysporium* isolate SH1 (**B**), Morphological under the light of *Fusarium oxysporium* isolate SH1 (**C**), fungus-mediated silver nanoparticles (AgNPs).

Three of the five fungal isolates that were isolated from infected tomato samples cultured on Czapek’s-Doxagar (CDA) medium were able to produce silver nanoparticles by green synthesis using their cell-free filtration. Universal keys were used to identify these fungal isolates. For additional research, the most potent fungal isolate with the highest yield of SNPs was chosen. First identified morphologically, this fungal isolate was subsequently identified genetically. Morphological identification revealed that fungal isolate SH1 appeared circular white cottony in color with a growing diameter of 30–40 mm as shown in (Fig. 1A). Additionally, “Microscopic observations revealed pigmented, smooth, multi-septate conidia that are cylindrical to slightly curved, with a characteristic central convex swelling.” As shown in (Fig. 1B) The reduction of silver ions using cell-free filtrates, known as septate hyphae, was first studied by comparing the color change to a pale purple hue. This revealed that different biomolecules, such as proteins, enzymes, polysaccharides, vitamins, and amino acids, were responsible for the reduction of Ag^+^^40,41^. Fungi can create a variety of bioactive compounds with a wide range of applications. Their capacity to withstand, absorb, and bioaccumulate heavy metals sets them apart from other bacteria^42^. They can be widely used in small-scale nanomaterials as stabilizing and capping agents. Microorganisms can be used for either intracellular or extracellular production of SNPs^43^. However, extracellular production of nanoparticles is favored over intracellular synthesis due to its simplicity, rapidity, and ease of purification. In the present study, extracellular biosynthesis was used as a traditional and beneficial technique. It was reported that the extracellular creation of silver nanoparticles utilizing a variety of microbes and discovered that incubation 1.0 mM AgNO_3_ with the cell-free supernatant caused the color to change from light yellow to pale purple. (Fig. 1C)

### Molecular identification amplification of 18 S rRNA

Regarding the selected isolate’s 18 S rRNA gene amplification. They were discovered to have the same PCR product size, recording roughly 600 bp, as indicated in **(Supplementary Fig.****S1****).**

### Construction of phylogenetic trees

**Fig. 2:**
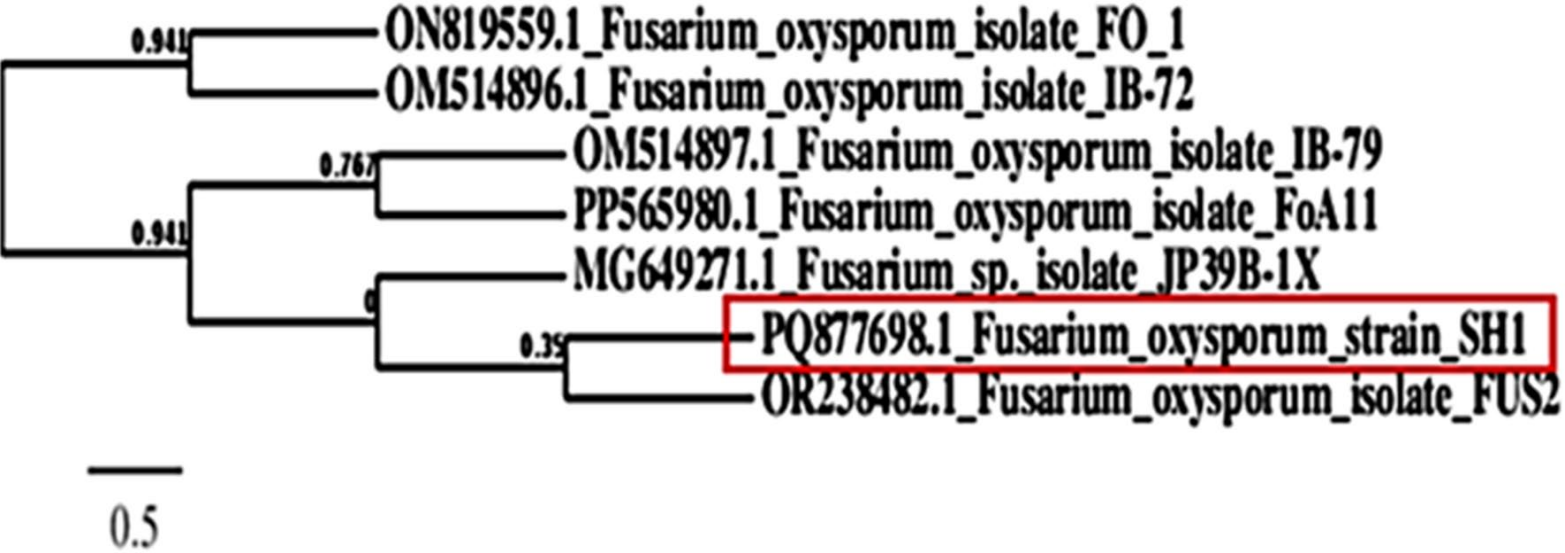
MUSLCE multiple sequence alignment program was used to do a phylogenetic analysis of the *F. oxysporum* SH1 *s*equence with nearby sequences from NCBI; the bar length for each nucleotide location indicates 0.5 substitutions.

The whole sequence of the internal transcribed sequence (ITS) of the rDNA region further confirmed morphological identification of *Fusarium oxysporum* SH1. Using the NCBI database **as** shown in (**Supplementary Table 1 (**BLAST search engine, *Fusarium oxysporum* was determined to be the amplicon. The phylogenetic tree was constructed using the ClustalW tool, as shown in **(**Fig. 2**).** The sequence was contributed to the database with accession number PQ877698.1 after achieving a 99.0% similarity rate to the isolates of *Fusarium oxysporum*, which had accession numbers ON819559.1, OM514896.1, OM514897.1, PP565980.1, MG649271.1, and OR238482.1. Additionally, it had 99% query coverage and zero E-value.

### Characterization of AgNPs

#### UV–Vis spectroscopy of AgNPs

**Fig. 3:**
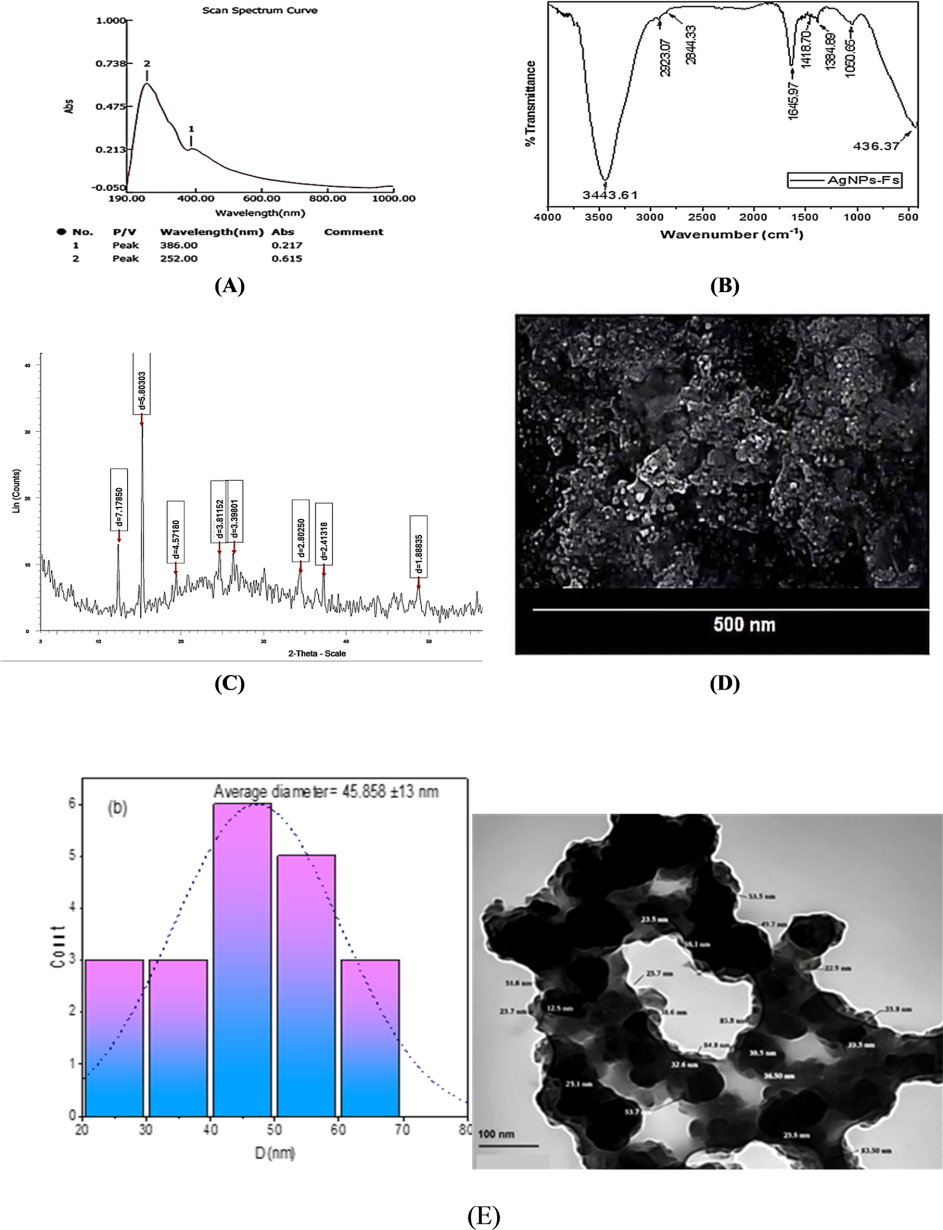

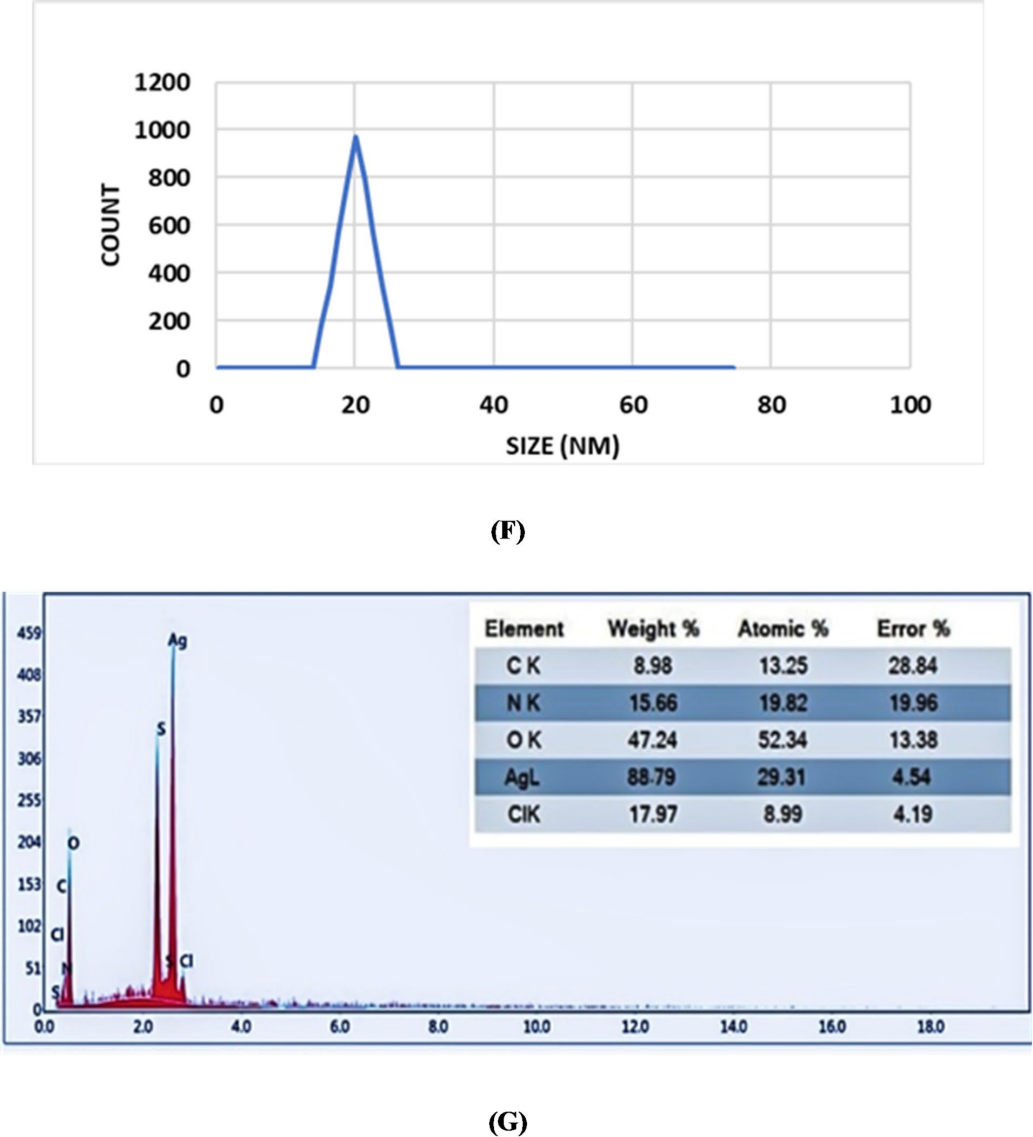
(**A**) UV-Vis spectrum. The apex of surface plasmon resonance of the AgNP-Fs solution observed at 386 nm confirmed the synthesis of small, polydisperse silver nanoparticles. (**B**) FTIR analysis of silver nanoparticles produced by *Fusarium* sp. (AgNPs-Fs). (**C**) XRD Analysis of Fusarium-Derived Silver Nanoparticles (AgNPs-Fs). (**D**) An illustration of silver nanoparticles (AgNPs-Fs) produced by scanning electron microscopy (SEM) *Fusarium oxysporium* SH1. (**E**) An illustration of the biosynthesized silver nanoparticles (AgNPs-Fs) using transmission electron microscopy (TEM) *by Fusarium oxysporium* SH1. (**F**) Dynamic Light Scattering (DLS) analysis of biosynthesized silver nanoparticles (AgNPs-Fs) using *Fusarium oxysporium* SH1. (**G**) The EDAX spectrum shows the elemental composition of silver nanoparticles (AgNPs-Fs) synthesized using *Fusarium oxysporum* SH1 *sp.*

Among the most popular methods was described for UV-Vis spectroscopy of silver nanoparticles^44^. Plasmon excitation of silver nanoparticles was indicated during cellular production by a single peak at 400 nm, as illustrated in **(**Fig. 3A**).** The metal nanoparticles’ surface plasmon excitation is the cause of several researchers who have found absorption of a liquid’s colloidal silver peak between 400 and 450 nm. According to^45^, Mie’s theory was used to simulate SPR. It is consistent with peaks at 400 and 420 nm.

#### Fourier-Transform Infrared (FTIR) Analysis

Several different vibrational bands that are indicative of functional groups involved in nanoparticle production and surface stability were seen in the FTIR spectrum of biosynthesized silver nanoparticles (AgNPs-Fs) generated by *Fusarium sp*. **(**Fig. 3B**).** O-H stretching vibrations, which may come from hydroxyl groups in alcohol or phenolic compounds and are implicated in the bioreduction of silver ions, are responsible for a broad absorption peak that is centered at 3443.61 cm^− 1^. The existence of lipid or hydrocarbon residues that may be connected to fungal metabolites is suggested by two additional peaks that are situated at 2923.07 cm^− 1^ and 2844.33 cm^− 1^. These peaks align with the C-H extending vibrations of aliphatic chains^46^. Peptides may aid in nanoparticle capping, as evidenced by the conspicuous band at 1645.97 cm^− 1^, which is attributed to the C = O stretching of amide I, a distinctive protein signature^47^.Additional peaks at 1418.70 cm-^1^ and 1384.89 cm^− 1^ are ascribed to C-N stretching and O-H bending vibrations, which are frequently present in carboxylate groups or amino acids, respectively^48^. According to^49^, a discernible absorption at 1050.65 cm^− 1^ is suggestive of stretching of C-O, perhaps from polysaccharides or ester groups. Crucially, the band seen at 436.37 cm^− 1^ is located in the low-frequency range frequently linked to metal-oxygen bonding^50^, demonstrating that silver ions and fungal proteins can successfully interact. This promotes the use of green synthesis to create stable silver nanoparticles. The spectrum as a whole demonstrates that many bioactive compounds released by *Fusarium*, including proteins, phenolics, and polysaccharides, help ensure that the resulting AgNPs are stable as well as the reduction of Ag^+^ ions^51^.

#### X-ray diffraction analysis

X-ray diffraction was used to examine the biosynthesized *Fusarium*-derived silver nanoparticles’ (AgNPs-Fs) crystalline nature and phase purity. analysis. )Fig. 3C **(**presents the XRD pattern of the AgNPs-Fs, revealing several distinctive diffraction peaks. The diffractogram exhibited well-defined peaks at 2θ values of 12.32°, 15.26°, 19.40°, 24.63°, 26.21°, 34.43°, 37.23° and 48.75°. Interestingly, this diffraction pattern deviates from the typical face-centered cubic (FCC) crystalline structure commonly observed in chemically synthesized silver nanoparticles, which usually displays characteristic peaks at 2θ values that correspond to the (111), (200), (220), and (311) planes, roughly 38°, 44°, 64°, and 77° .This variation implies that a distinct crystallographic structure has been produced by fungal-mediated biosynthesis, Perhaps because of the way silver ions interact with one another and different biomolecules released by *Fusarium sp*. during the bioreduction process^52^. With a relative intensity of 100%, the strongest diffraction peak was detected at 2θ = 15.26° (d = 5.80 Å). There were three other notable peaks at 12.32° (40.5%), 24.63° (38.2%), and 26.21° (36.6%) with relative intensities greater than 35%. The existence of many well-defined peaks demonstrates the polycrystalline nature of the biosynthesized AgNPs-Fs^53^. The Debye-Scherrer equation applied to the average crystallite size of the AgNPs-Fs was estimated using the diffraction peaks. The estimated crystallite sizes for each peak seen in **(**Table 1**)** are displayed in the XRD pattern.

**Table 1:** Crystallite sizes of AgNPs-Fs computed using the Debye-Scherrer equation from XRD peaks.

Peak (2θ°)	d-spacing (Å)	Relative intensity (%)	FWHM (β)*	Crystallite size (nm)**
12.323	7.17656	40.5	0.28	28.5
15.256	5.80303	100	0.32	25.2
19.400	4.57189	27.8	0.35	23.2
24.630	3.61152	38.2	0.38	21.6
26.205	3.39801	36.6	0.01	48.2
34.432	2.60260	28.4	0.45	18.8
37.230	2.41318	30.5	0.47	18.1
48.753	1.86635	23.2	0.52	17.1

FWHM* values are calculated using the typical peak broadening found in samples of nanoparticles determined using the Debye-Scherrer equation, where λ = 1.542 Å and D = 0.9λ/βcosθ .The successful biogenesis of the average crystallite size was calculated to validate the presence of silver nanoparticles across all peaks, which was 25.08 ± 3.8 nm, falling inside the nanoscale range^54^. Effective control over nucleation during the fungal-mediated synthesis process is shown by the comparatively modest crystallite size. Additionally, the excellent purity of the biosynthesized AgNPs-Fs is indicated by the lack of additional peaks, indicating that any crystalline impurities were successfully removed throughout the purification process. Even though biological entities were involved in the synthesis process, the well-defined and somewhat sharp diffraction peaks also indicate that the biosynthesized nanoparticles have good crystallinity^55^.

#### SEM morphological investigation

SEM was utilized to assess the surface morphology and approximate visual distribution of the silver nanoparticles generated by *Fusarium sp*. The findings are displayed in **(**Fig. 3D**).** A dense cluster of spherical nanoparticles with a largely consistent size distribution and no discernible elongation or distortion was visible in the micrograph. Perhaps as a result of the drying process or weak van der Waals forces, the particles seemed to be evenly distributed in clusters. Fungal metabolites, which function as stabilizing and reducing agents, are frequently implicated in extracellular biosynthetic processes that lead to the creation of spherical forms^56^. Natural drying or leftover biomolecules that were not completely eliminated during purification could be the cause of the small aggregation that is observed.

#### TEM analysis (AgNPs-Fs)

Transmission electron microscopy (Fig. 3E) revealed that the biosynthesized silver nanoparticles were retained near-spherical with relatively uniform morphology^57^. The particle diameters ranged from 35 to 50 nm, with an average size of approximately 45.8 nm. Minor variations in shape were observed in a small proportion of particles^58^. The contrast between particle cores and surrounding regions may indicate the presence of biological capping materials formed during extracellular synthesis^59^.

#### Dynamic Light Scattering (DLS)

According to the DLS analysis, the average hydrodynamic diameter of the silver nanoparticles produced by *Fusarium sp.* was roughly 20.1 nm, as shown in **(**Fig. 3F**).** The biosynthetic method produced comparatively homogenous nanoparticles with little aggregation, as evidenced by the narrow size distribution. It is crucial to remember that due to the existence of a hydration shell or surface-bound proteins, the hydrodynamic diameter determined by DLS typically tends to be somewhat greater than the core size determined by microscopic methods. The uniformity of size indicates how well the fungal metabolites stabilize the creation of nanoparticles throughout biosynthesis. Because of these size properties, AgNPs-Fs are attractive options for biomedical applications, where cellular uptake and interaction depend heavily on nanoscale dimensions^60^.

#### EDAX analysis

The EDAX spectrum of the biosynthesized AgNPs-Fs revealed a strong signal corresponding to silver (Ag), with a prominent peak at approximately 3 keV, which is characteristic of metallic silver nanoparticles **(**Fig. 3G**).** Silver was the main element, according to quantitative analysis, with an atomic percentage of 88.79% and a weight% of 29.31%, confirming the successful formation of silver nanoparticles. In addition to silver, peaks corresponding to oxygen (47.24 wt%), nitrogen (15.66 wt%), carbon (8.98 wt%), sulfur (minor traces), and chlorine (17.97 wt%) were also detected. The presence of C, N, and O is attributed to the organic biomolecules (proteins, polysaccharides, and other metabolites) secreted by *Fusarium oxysporium* SH1, which serve as stabilizing and reducing agents during the production of nanoparticles^61^. These biomolecules are responsible for capping the nanoparticles, thereby enhancing their stability and preventing aggregation. The detection of chlorine may be related to residual salts from the culture medium or the fungal metabolites, while the small sulfur peak may indicate the involvement of sulfur-containing amino acids in the reduction process^62^. Similar studies have reported the coexistence of silver with carbon, oxygen, and nitrogen peaks in biosynthesized nanoparticles, supporting Fungal-derived metabolites’ function in nanoparticle stabilization^63^. The strong silver signal observed in this study, combined with the presence of capping elements, confirms both the high purity of the nanoparticles and the effectiveness of the green synthesis method employed.

#### Molecular identification of the multidrug-resistant bacteria

**Fig. 4:**
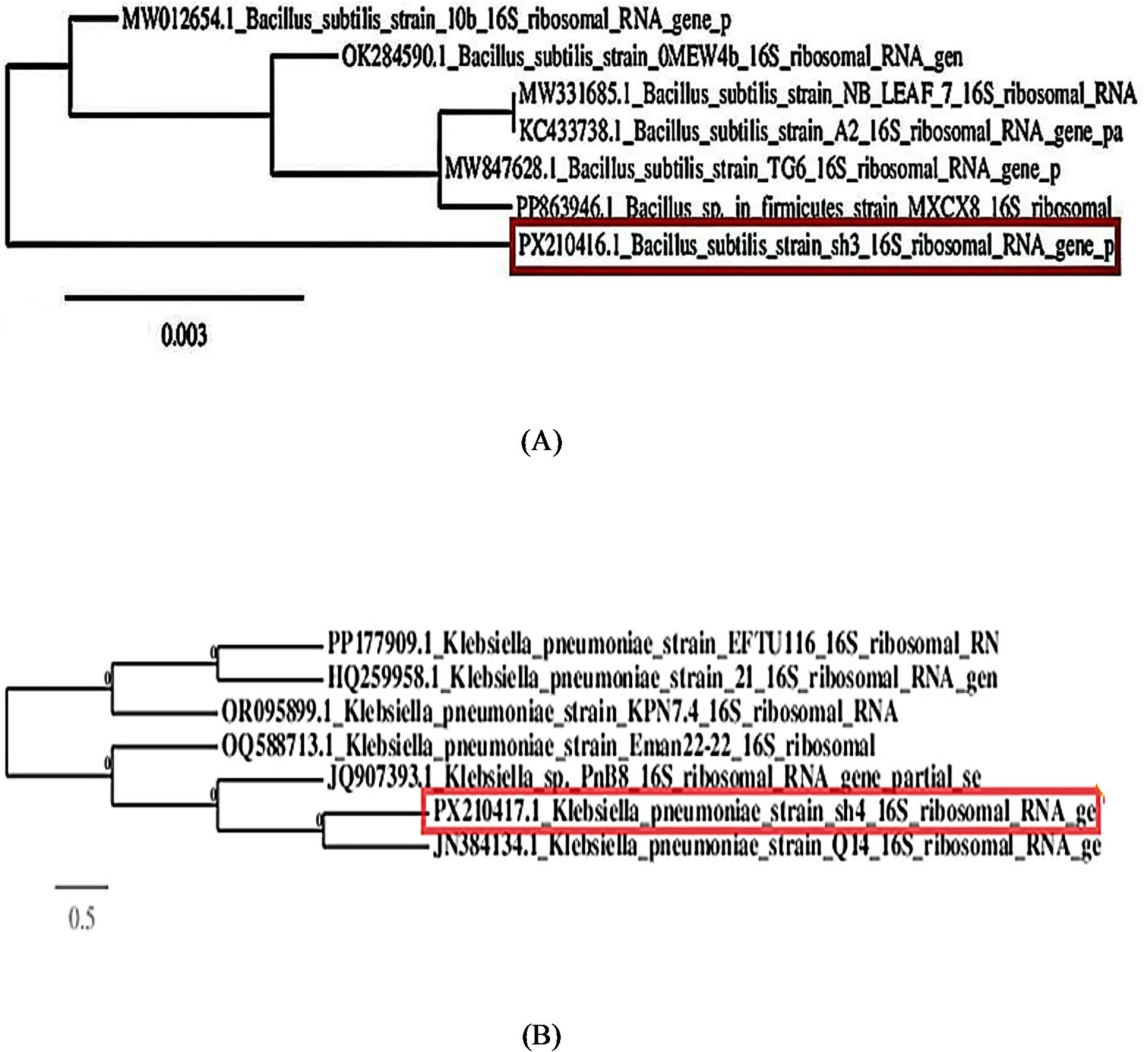
(**A**) Phylogenetic analyses of *Bacillus subtilis strain* the 16 S rRNA region were amplified and sequenced to identify sh3 bacteria. Using the ClustalW multiple sequence alignment program, a phylogenetic tree was constructed using the NCBI closely related sequences. Each nucleotide location has 0.003 substitutions shown by the bar length. (**B**) Phylogenetic analyses of *Klebsiella pneumoniae* sh4 bacteria according to the 16 S rRNA region’s amplification and sequencing. Using the NCBI closely related sequences, a phylogenetic tree was constructed. Each nucleotide location has 0.5 substitutions shown by the bar length.

Two bacterial isolates were chosen and identified using 16 S rRNA gene sequencing. Purification, sequencing, and analysis of the two isolates’ 16 S rRNA gene amplifications were performed on the amplicons sh3 and sh4. A non-redundant BLAST search revealed that they were equal in terms of the size of their PCR result, which recorded approximately 1485 bp, as seen in **(Supplementary Fig.****S2****).** The accession codes **PX210416.1 and PX210417.1** were assigned to the 16 S rRNA genes that were partially sequenced and subsequently presented to the GenBank database. Closely similar 16 S rRNA sequences from GenBank were used to create phylogenetic trees. Multidrug-resistant bacteria’s susceptibility to SNPs: As illustrated in Fig. 4A, B, the antimicrobial activity of SNPs made from *F. oxysporum* cell extract was evaluated against two multidrug-resistant bacteria, one of which was Gram-positive (*Bacillus subtilis* sh3) and the other Gram-negative (*Klebsiella pneumoniae* sh4).

### Antimicrobial evaluation of fusarium AgNPs

**Fig. 5:**
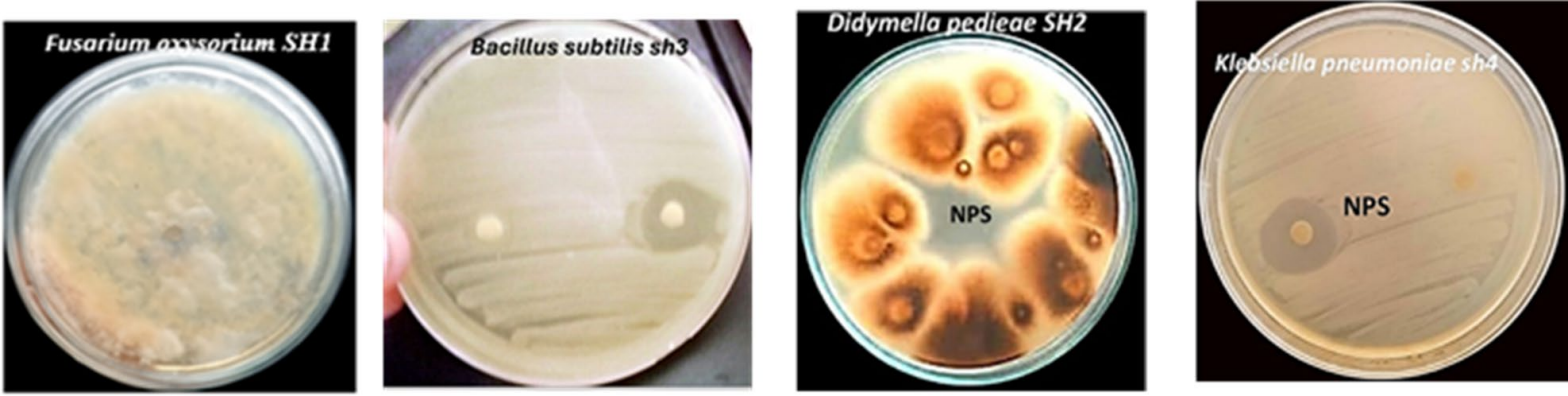
Antimicrobial efficiency of AgNPs produced from *F. oxysporium* SH1 isolate PQ877698.1 against gram-positive, gram negative, and fungi.

Using information in the disc diffusion agar method **(**Fig. 5**)** demonstrates the antimicrobial efficacy of AgNPs against microbes containing fungal isolates and both gram-positive and gram-negative bacteria. According to the study, AgNPs were effective against both gram-positive and gram-negative bacteria as well as fungi. *F. oxysorium* SH1 showed no effect, while the inhibitory zones of *B. subtilis* sh3, *Klebsiella pneumoniae* sh4 and *Didymella pedieae* were roughly 12.5, 13.5, and 19.5 mm in size, respectively.

### Estimation of synergistic antimicrobial activity

#### Synergistic activity of AgNPs with antibiotics

**Fig. 6:**
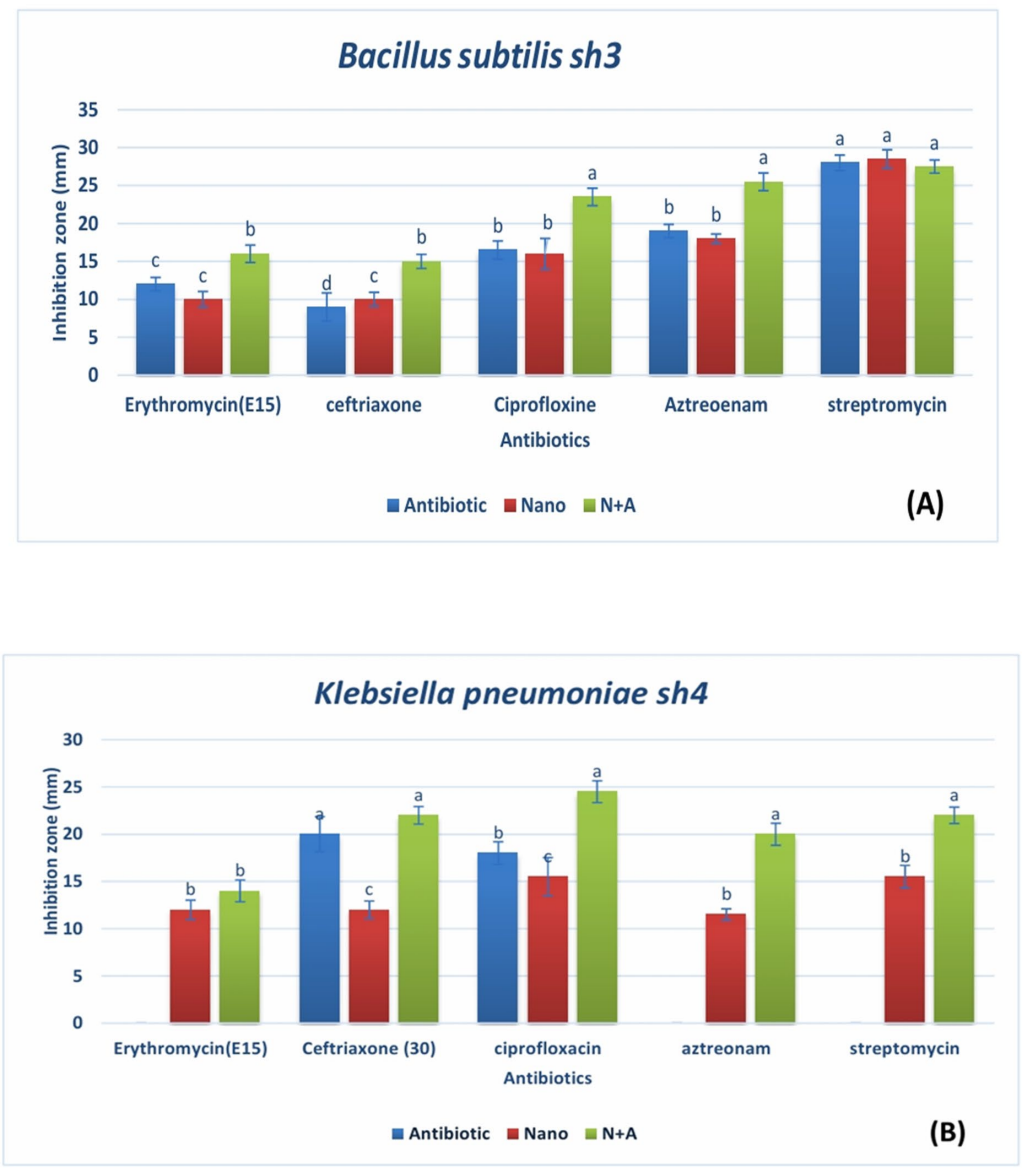
Antibacterial activity (inhibition zone, mm) of the SNPs synthesized using the extract of *Fusarium oxysporum* SH1 against (**A**) *Bacillus subtilis* sh3 and (**B**) *Klebsiella pneumoniae* sh4. Different letters (a, b, c, d) indicate statistically significant differences at *p* < 0.05.

The antibacterial efficacy of biologically produced AgNPs in conjunction with antibiotics was examined against *B. subtilis* sh3 and *K. pneumoniae* sh4. Notably, combining AgNPs with selected antibiotics resulted in enlarged inhibition zones compared to individual treatments, indicating a potential synergistic interaction. The observed differences in inhibition zone diameters were statistically confirmed, where treatments bearing different letters were regarded as significantly distinct at *p* < 0.05. In contrast to antibiotics or AgNPs alone, the inclusion of AgNPs typically increased the inhibition zone widths, as shown in supplementary Tables 2 and supplementary Table 3 file suggesting a potential impact. The combined treatment considerably appears to increase the antibacterial activity of Erythromycin, Ceftriaxone, Ciprofloxacin, and Aztreonam *against B. subtilis* sh3, but streptomycin only showed a minor improvement. When coupled with AgNPs, ciprofloxacin and aztreonam showed the greatest increase in inhibitory zone diameter **(**Fig. 6A**).** It was believed that the antagonistic effect of the biological synthesis of AgNPs was demonstrated by the decrease or increase in the width of the inhibitory zone surrounding the various antibiotic disks (E, AT, CTR, S, and CIP) in conjunction with AgNPs synthesis. The presence of a zone of inhibition indicated the antibacterial effectiveness of AgNPs coupled with antibiotic disk. The disk diffusion assay indicated that the size of the inhibitory zone varied depending on the type of bacteria^64^. The inhibitory zone results for five distinct antibiotic kinds employing biological AgNPs synthesis against two dangerous bacterial species, *Bacillus subtilis* sh3 *and Klebsiella pneumoniae* sh4, are shown in Fig. 6A **and B.**

### Determination of Minimum Inhibitory Concentration (MIC) of AgNPs-Fs

**Fig. 7:**
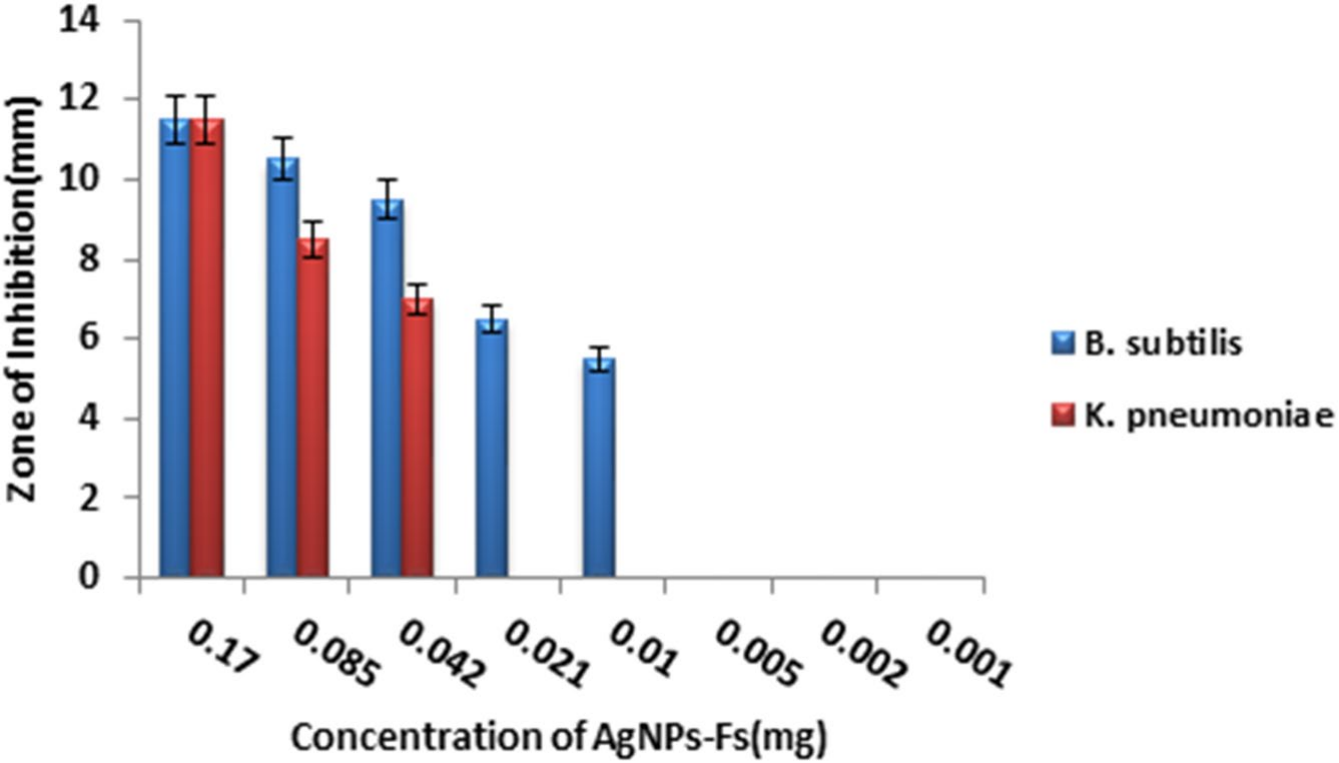
Minimum inhibitory concentration value of silver nanoparticles synthesized via *Fusarium* sp. (AgNPs-Fs) against *Bacillus subtilis* sh3 and *Klebsiella pneumoniae* sh4, showing inhibition zone diameters at different nanoparticle concentrations.

The biosynthesized AgNPs-Fs displayed a clear concentration-dependent antibacterial effect (Fig. 7). At 0.17 mg/mL, the inhibition zones reached 12.0 ± 0.5 mm for *B. subtilis* sh3 and 11.5 ± 0.6 mm for *K. pneumoniae* sh4. The MIC values were determined to be 0.005 mg/mL for *B. subtilis* and 0.01 mg/mL for *K. pneumoniae* (Table 2). These values are significantly lower than those reported in previous studies, where MICs of biosynthesized AgNPs generally ranged between 0.05 and 0.25 mg/mL for Gram-positive strains and 0.1–0.5 mg/mL for Gram-negative strains^65^. The enhanced activity observed in this work underscores the superior antibacterial activity of *F. oxysporum*–derived AgNPs, likely due to their smaller size and effective biomolecular capping.

**Table 2:** Minimum inhibitory concentration (MIC) and inhibition zones of biosynthesized silver nanoparticles (AgNPs-Fs) against *Bacillus subtilis* sh3 and *Klebsiella pneumoniae* sh4.

Bacterial strain	MIC (mg/mL)	Inhibition zone (mm)
*Bacillus subtilis* sh3	0.005	12.0 ± 0.5
*Klebsiella pneumoniae* sh4	0.01	11.5 ± 0.6

### Cytotoxicity evaluation

**Fig. 8:**
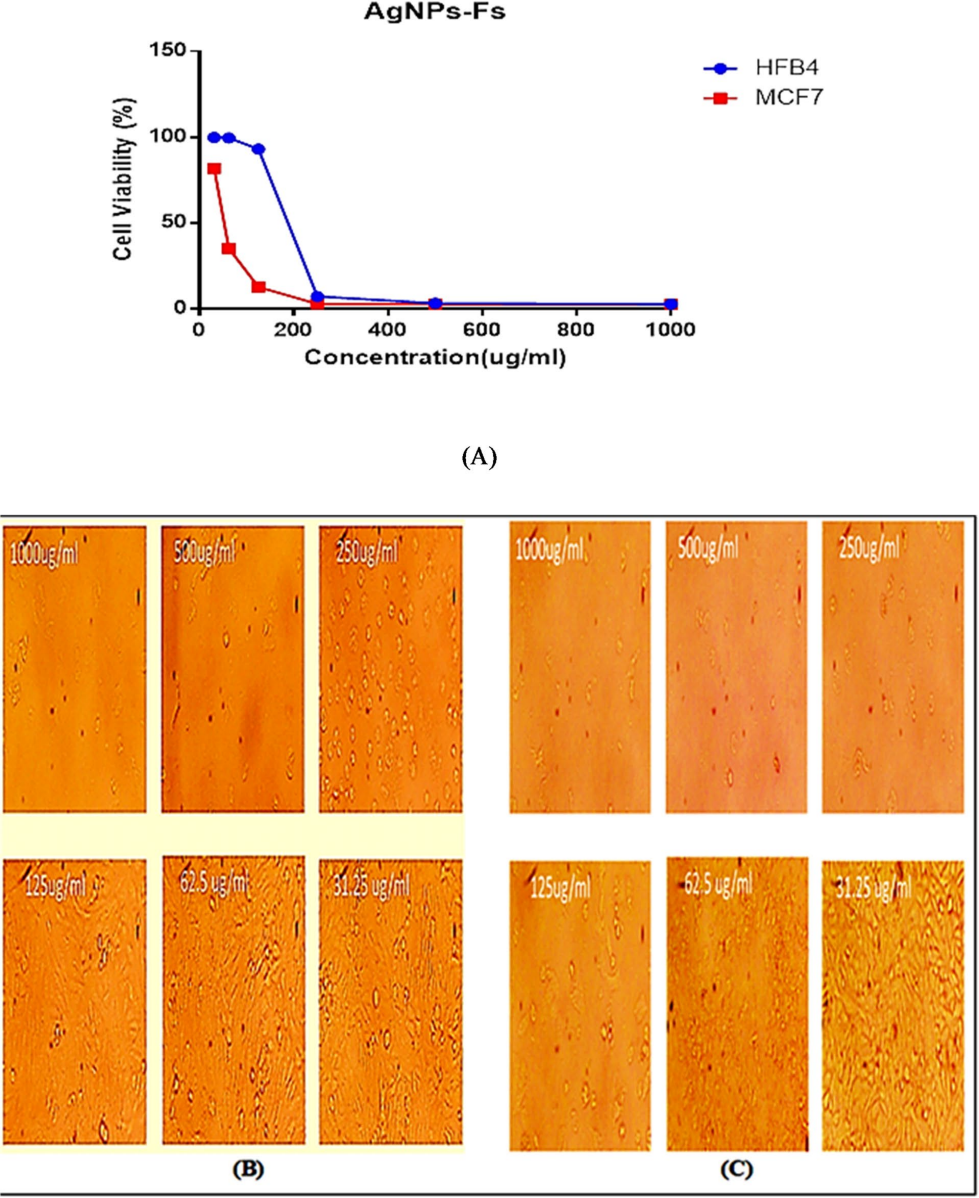
(**A**) Cytotoxic effect of biosynthesized silver nanoparticles from *F. oxysporium* SH1 (AgNPs-Fs) on MCF7 breast cancer cells and HFB4 normal cells as assessed by the MTT assay. Representative microscopic images showing the morphological changes induced by biosynthesized silver nanoparticles (**B**) HFB4 normal cells treated with (AgNPs-Fs) and (**C**) MCF7 breast cancer cells treated with AgNPs-Fs.

The MTT experiment showed that silver nanoparticles produced by Fusarium sp. (AgNPs-Fs) inhibit MCF7 breast cancer cells in a way that is dependent on dosage. Normal HFB4 melanocytes had a much greater IC_50_ of 171.63 µg/mL **(**Table 3**)** than MCF7 cells, which had an IC_50_ of 64.34 µg/mL^66^. This difference produced a selectivity index (SI) of 2.67, which indicates moderate selectivity. Accordingly, the statement regarding selective growth inhibition has been moderated to describe preferential cytotoxicity towards MCF7 cells. Morphological observations from phase-contrast microscopy are included to provide supporting evidence for differential effects on cancer versus normal cells. Because of their higher inhibitory effects on cancer cells and comparatively lesser toxicity on normal counterparts, AgNPs-Fs remain a promising candidate for further investigation. Interestingly, the SH1-derived nanoparticles demonstrated slightly higher selectivity and cytotoxic efficiency compared to what has been reported for other *F. oxysporum* strains, indicating a unique biological advantage of this strain^67^. The dose-response profiles **(**Fig. 8A**)** make it evident that MCF7 cells are more susceptible than HFB4 cells, as they showed a more pronounced drop in viability as the quantity of nanoparticles increased. This increased sensitivity might result from cancer cells’ heightened metabolic activity and weakened oxidative stress defense systems, which make them more vulnerable to cytotoxic stress brought on by nanoparticles. These outcomes are consistent with earlier research on the anticancer effectiveness of biosynthesized silver nanoparticles, which found that increased uptake of the nanoparticles, The primary reasons of selective toxicity were elevated reactive oxygen species (ROS) generation and mitochondrial dysfunction in tumor cells^68^.

### Morphological alterations

After being exposed to AgNPs-Fs, MCF7 cells showed significant morphological alterations, including rounding, shrinkage, separation from the culture surface, and monolayer fragmentation, as seen by phase-contrast microscopy **(**Fig. 8C**)**^69^. Conversely, at comparable.

concentrations, HFB4 cells displayed fewer structural changes; although, at higher levels, some rounding and decreased adherence were noted **(**Fig. 8B**).** These morphological results align with previously documented necrotic and apoptotic pathways in tumor cells treated with nanoparticles^70^. Numerous processes, such as DNA damage, mitochondrial malfunction, and reactive oxygen species (ROS) production, have been linked to the observed cytotoxicity in silver nanoparticle-mediated cancer therapy^71^. Malignant and non-malignant cells may differ in their cellular absorption and metabolic responses, which could account for the selective toxicity toward MCF7 cells^72^.

**Table 3:** IC_50_ and Selectivity Index (SI) of silver nanoparticles from *Fusarium* (AgNPs-Fs) on MCF7 breast cancer cells and HFB4 normal cells.

Samples	IC_50_ (µg/mL) MCF7	IC_50_ (µg/mL) HFB4	Selectivity Index (SI)
AgNPs-Fs (SH1)	64.34	171.63	2.67

## Conclusion

This study shows that the extracellular manufacture of silver nanoparticles may be carried out effectively and sustainably using *Fusarium oxysporum* SH1 as a biological system. The crystalline structure, functional capping by fungal metabolites, and nanoscale dimensions of the biosynthesized AgNPs were all well-characterized. These nanoparticles demonstrated notable antibacterial activity against multidrug-resistant *Bacillus subtilis* sh3 *and Klebsiella pneumoniae* sh4, and they appeared to enhance antimicrobial efficacy through potentiated interactions when used with traditional medicines. The AgNPs’ therapeutic potential was further demonstrated by their specific cytotoxicity toward breast cancer (MCF7) cells and relatively minimal toxicity toward healthy melanocytes. When combined, the results demonstrate that fungal-mediated AgNPs have both antibacterial and anticancer properties and point to their potential as environmentally benign nanomaterials for use in pharmaceutical and biological applications. This biosynthetic approach is environmentally favorable, as it does not involve hazardous chemical reducing agents and proceeds under mild reaction conditions with minimal waste generation. Their molecular mechanisms of action, large-scale production optimization, and in vivo safety validation should all be the focus of future research.

## Supplementary Information

Below is the link to the electronic supplementary material.

Supplementary Material 1

Supplementary Material 2

Supplementary Material 3

Supplementary Material 4

## Data Availability

The datasets generated and/or analyzed during the current study are available in the GenBank NCBI repository link: http://www.ncbi.nlm.nih.gov/ and the accession numbers are:1. NCBI Gene Bank Accession No: PQ877698.12. NCBI Gene Bank Accession No **:** PQ877698"3. NCBI Gene Bank Accession No: PX210416.14. NCBI Gene Bank Accession No: PX210416"5.NCBI Gene Bank Accession No: PX210417.16.NCBI Gene Bank Accession No: PX210417”.
