# Supplementary material for: Molecular identification, characterization and antibacterial activity of fungal-mediated silver nanoparticles against Bacillus subtilis sh3 and Klebsiella pneumoniae sh4
Source: Sci Rep. 2026 Mar 29;16:10728. doi: 10.1038/s41598-026-42107-9 (PMC13039263; doi:10.1038/s41598-026-42107-9)
Supplement: Supplementary file 1 — Supplementary Material 1 [file 41598_2026_42107_MOESM1_ESM.docx]

**Support file**


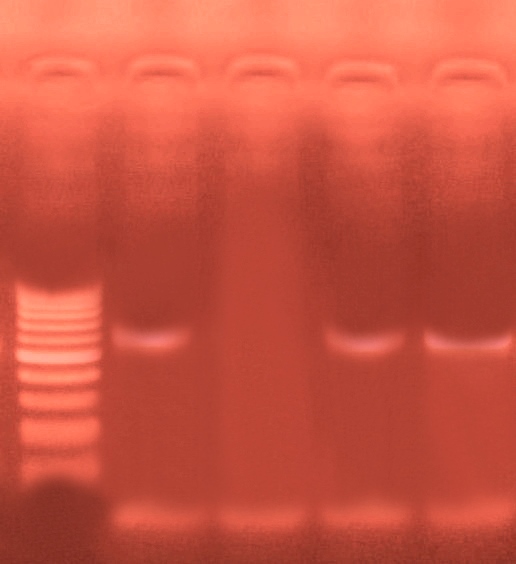


**Fig. 3**: The fungus strains' amplified 18s rRNA PCR products are displayed on an agarose gel electrophoresis. (*SH1)*.


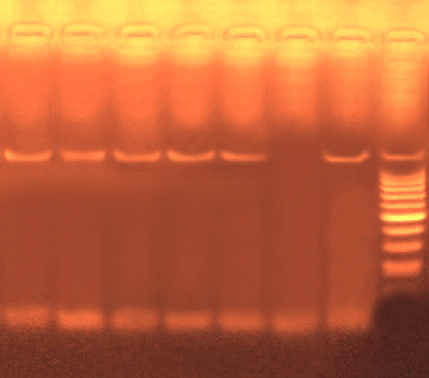


**Fig.12:** Agarose gel electrophoresis shows the amplified 16s rRNA PCR products from the

isolated bacterial strains. (sh3 and sh4).
