# Supplementary material for: Molecular identification, characterization and antibacterial activity of fungal-mediated silver nanoparticles against Bacillus subtilis sh3 and Klebsiella pneumoniae sh4
Source: Sci Rep. 2026 Mar 29;16:10728. doi: 10.1038/s41598-026-42107-9 (PMC13039263; doi:10.1038/s41598-026-42107-9)
Supplement: Supplementary file 3 — Supplementary Material 3 [file 41598_2026_42107_MOESM3_ESM.docx]

**Supplementary file**

 **Supplementary Table 1: Top** 9 hits description on the query of Sanger sequence 18s rRNA *Fusarium oxysporum* strain SH1 on BLAST

**Supplementary Fig.S1:** The fungus strains' amplified 18s rRNA PCR products are displayed on an agarose gel electrophoresis. (*SH1)*.

**Supplementary Fig.S2:** Agarose gel electrophoresis shows the amplified 16s rRNA PCR products from the isolated bacterial strains. (sh3 and sh4)

**Supplementary Table 2:** Antibacterial activity of antibiotics, AgNPs, and their combinations against *Bacillus subtilis* sh3*.*

|  | ***Bacillus subtilis sh3*** | | | |
| --- | --- | --- | --- | --- |
| **Antibiotics** | **control** | **Antibiotic(mm)** | **Nano(mm)** | **N+A(mm)** |
| **Erythromycin(E15)** | **0** | **12± 1.03** | **12.5 ± 0.29** | **16± 1.00** |
| **Ceftriaxone** | **0** | **9 ± 1.11** | **12.5± 0.29** | **15± 1.00** |
| **Ciprofloxine** | **0** | **16.5± 1.12** | **12.5± 0.29** | **23.5 ± 0.76** |
| **Aztreoenam** | **0** | **19± 1.01** | **12.5± 0.29** | **25.5 ± 1.04** |
| **Streptromycin** | **0** | **28± 1.14** | **12.5± 0.29** | **27.5 ± 1.04** |

**Supplementary Table 3:** Antibacterial activity of antibiotics, AgNPs, and their combinations against *Klebsiella pneumoniae sh4.*

|  | ***Klebsiella pneumoniae sh4*** | | | |
| --- | --- | --- | --- | --- |
| **Antibiotics** | **control** | **Antibiotic(mm)** | **Nano(mm)** | **N+A(mm)** |
| **Erythromycin(E15)** | **0** | **0 ±1.10a** | **19.5 ± 0.50** | **14 ± 1.00** |
| **Ceftriaxone (30)** | **0** | **20 ±1.11** | **19.5 ± 0.50** | **22 ± 1.00** |
| **Ciprofloxacin** | **0** | **18 ±1.12** | **19.5 ± 0.58** | **24.5 ± 0.76** |
| **Aztreonam** | **0** | **0 ±1.10** | **19.5 ± 0.58** | **20 ± 1.00** |
| **Streptomycin** | **0** | **0 ±1.10** | **19.5 ± 0.50** | **22 ± 1.00** |
